# Supplementary material for: Development of a computer-aided design software for the quantitative evaluation of aesthetic damage
Source: PLoS One. 2019 Dec 18;14(12):e0226322. doi: 10.1371/journal.pone.0226322 (PMC6919621; doi:10.1371/journal.pone.0226322)
Supplement: S1 Code site — 1. (ZIP) [file pone.0226322.s001.zip › [EESC_jr][Projeto]Relatorio_07_21/reports/templates_zip/gentelella-master.zip!/gentelella-master/vendors/DateJS/reports/lcov-report/core/extras.js.html]

Code coverage report for core/extras.js


# Code coverage report for core/extras.js

## Statements: 61.76% (42 / 68)      Branches: 33.33% (15 / 45)      Functions: 92.31% (12 / 13)      Lines: 61.76% (42 / 68)      Ignored: none

All files » core/ » extras.js

```
|  |  |  |
| --- | --- | --- |
| 1 2 3 4 5 6 7 8 9 10 11 12 13 14 15 16 17 18 19 20 21 22 23 24 25 26 27 28 29 30 31 32 33 34 35 36 37 38 39 40 41 42 43 44 45 46 47 48 49 50 51 52 53 54 55 56 57 58 59 60 61 62 63 64 65 66 67 68 69 70 71 72 73 74 75 76 77 78 79 80 81 82 83 84 85 86 87 88 89 90 91 92 93 94 95 96 97 98 99 100 101 102 103 104 105 106 107 108 109 110 111 112 113 114 115 116 117 118 119 120 121 122 123 124 125 126 127 128 129 130 131 132 133 134 135 136 137 138 139 140 141 142 143 144 145 146 147 148 149 150 151 152 153 154 155 156 157 158 159 160 161 162 163 164 165 166 167 168 169 170 171 172 173 174 175 176 177 178 179 180 181 182 183 184 185 186 187 188 189 190 191 192 193 194 195 196 197 198 199 200 201 202 203 204 205 206 207 208 209 210 211 212 213 214 215 216 217 218 219 220 221 222 223 224 225 226 227 228 229 230 231 232 233 234 235 236 237 238 239 240 241 242 243 244 245 246 247 248 249 250 251 252 253 254 255 256 257 258 259 260 261 262 263 264 265 266 267 268 269 270 271 272 273 274 275 276 277 278 279 280 281 282 283 284 285 286 287 288 289 290 291 292 293 294 295 296 297 298 299 300 301 302 303 304 305 306 307 308 309 310 311 312 313 314 | 1 1                                                       1                                                                                                     1   166     81 81                                                                             1                   6       6       166 166 166 85   81   81 1   80         1 8 166                           1 2 2                           1 2 2                                                                                                                                                                                             1 3 6 6       6 6 6 6       1 3 3 1   2       1 1 | ``` (function () { 	var $D = Date, 		$P = $D.prototype, 		// $C = $D.CultureInfo, // not used atm 		p = function (s, l) { 			if (!l) { 				l = 2; 			} 			return ("000" + s).slice(l * -1); 		}; 	/** 	 * Converts a PHP format string to Java/.NET format string.  	 * A PHP format string can be used with ._format or .format. 	 * A Java/.NET format string can be used with .toString(). 	 * The .parseExact function will only accept a Java/.NET format string 	 * 	 * Example 	 * var f1 = "%m/%d/%y" 	 * var f2 = Date.normalizeFormat(f1);	// "MM/dd/yy" 	 *  	 * new Date().format(f1);	// "04/13/08" 	 * new Date()._format(f1);	// "04/13/08" 	 * new Date().toString(f2);	// "04/13/08" 	 *   	 * var date = Date.parseExact("04/13/08", f2); // Sun Apr 13 2008 	 *  	 * @param {String}   A PHP format string consisting of one or more format spcifiers. 	 * @return {String}  The PHP format converted to a Java/.NET format string. 	 */ 	 var normalizerSubstitutions = { 		"d" : "dd", 		"%d": "dd", 		"D" : "ddd", 		"%a": "ddd", 		"j" : "dddd", 		"l" : "dddd", 		"%A": "dddd", 		"S" : "S", 		"F" : "MMMM", 		"%B": "MMMM", 		"m" : "MM", 		"%m": "MM", 		"M" : "MMM", 		"%b": "MMM", 		"%h": "MMM", 		"n" : "M", 		"Y" : "yyyy", 		"%Y": "yyyy", 		"y" : "yy", 		"%y": "yy", 		"g" : "h", 		"%I": "h", 		"G" : "H", 		"h" : "hh", 		"H" : "HH", 		"%H": "HH", 		"i" : "mm", 		"%M": "mm", 		"s" : "ss", 		"%S": "ss", 		"%r": "hh:mm tt", 		"%R": "H:mm", 		"%T": "H:mm:ss", 		"%X": "t", 		"%x": "d", 		"%e": "d", 		"%D": "MM/dd/yy", 		"%n": "\\n", 		"%t": "\\t", 		"e" : "z", 		"T" : "z", 		"%z": "z", 		"%Z": "z", 		"Z" : "ZZ", 		"N" : "u", 		"w" : "u", 		"%w": "u", 		"W" : "W", 		"%V": "W", 	}; 	var normalizer = { 		substitutes: function (m) { 			return normalizerSubstitutions[m]; 		}, 		interpreted: function (m, x) { 			var y; 			switch (m) { 				case "%u": 					return x.getDay() + 1; 				case "z": 					return x.getOrdinalNumber(); 				case "%j": 					return p(x.getOrdinalNumber(), 3); 				case "%U": 					var d1 = x.clone().set({month: 0, day: 1}).addDays(-1).moveToDayOfWeek(0), 						d2 = x.clone().addDays(1).moveToDayOfWeek(0, -1); 					return (d2 < d1) ? "00" : p((d2.getOrdinalNumber() - d1.getOrdinalNumber()) / 7 + 1);   				case "%W": 					return p(x.getWeek()); 				case "t": 					return $D.getDaysInMonth(x.getFullYear(), x.getMonth()); 				case "o": 				case "%G": 					return x.setWeek(x.getISOWeek()).toString("yyyy"); 				case "%g": 					return x._format("%G").slice(-2); 				case "a": 				case "%p": 					return t("tt").toLowerCase(); 				case "A": 					return t("tt").toUpperCase(); 				case "u": 					return p(x.getMilliseconds(), 3); 				case "I": 					return (x.isDaylightSavingTime()) ? 1 : 0; 				case "O": 					return x.getUTCOffset(); 				case "P": 					y = x.getUTCOffset(); 					return y.substring(0, y.length - 2) + ":" + y.substring(y.length - 2); 				case "B": 					var now = new Date(); 					return Math.floor(((now.getHours() * 3600) + (now.getMinutes() * 60) + now.getSeconds() + (now.getTimezoneOffset() + 60) * 60) / 86.4); 				case "c": 					return x.toISOString().replace(/\"/g, ""); 				case "U": 					return $D.strtotime("now"); 				case "%c": 					return t("d") + " " + t("t"); 				case "%C": 					return Math.floor(x.getFullYear() / 100 + 1); 			} 		}, 		shouldOverrideDefaults: function (m) { 			switch (m) { 				case "%e": 					return true; 				default: 					return false; 			} 		}, 		parse: function (m, context) { 			var formatString, c = context \|\| new Date(); 			formatString = normalizer.substitutes(m); 			if (formatString) { 				return formatString; 			} 			formatString = normalizer.interpreted(m, c);   			if (formatString) { 				return formatString; 			} else { 				return m; 			} 		} 	};   	$D.normalizeFormat = function (format, context) { 		return format.replace(/(%\|\\)?.\|%%/g, function(t){ 				return normalizer.parse(t, context); 		}); 	}; 	/** 	 * Format a local Unix timestamp according to locale settings 	 *  	 * Example: 	 * Date.strftime("%m/%d/%y", new Date());		// "04/13/08" 	 * Date.strftime("c", "2008-04-13T17:52:03Z");	// "04/13/08" 	 *  	 * @param {String}   A format string consisting of one or more format spcifiers [Optional]. 	 * @param {Number\|String}   The number representing the number of seconds that have elapsed since January 1, 1970 (local time).  	 * @return {String}  A string representation of the current Date object. 	 */ 	$D.strftime = function (format, time) { 		var d = Date.parse(time); 		return d._format(format); 	}; 	/** 	 * Parse any textual datetime description into a Unix timestamp.  	 * A Unix timestamp is the number of seconds that have elapsed since January 1, 1970 (midnight UTC/GMT). 	 *  	 * Example: 	 * Date.strtotime("04/13/08");				// 1208044800 	 * Date.strtotime("1970-01-01T00:00:00Z");	// 0 	 *  	 * @param {String}   A format string consisting of one or more format spcifiers [Optional]. 	 * @param {Object}   A string or date object. 	 * @return {String}  A string representation of the current Date object. 	 */ 	$D.strtotime = function (time) { 		var d = $D.parse(time); 		return Math.round($D.UTC(d.getUTCFullYear(), d.getUTCMonth(), d.getUTCDate(), d.getUTCHours(), d.getUTCMinutes(), d.getUTCSeconds(), d.getUTCMilliseconds()) / 1000); 	}; 	/** 	 * Converts the value of the current Date object to its equivalent string representation using a PHP/Unix style of date format specifiers. 	 * Format Specifiers 	 * Format  Description																	Example 	 * ------  ---------------------------------------------------------------------------	----------------------- 	 * %a		abbreviated weekday name according to the current localed					"Mon" through "Sun" 	 * %A		full weekday name according to the current localed							"Sunday" through "Saturday" 	 * %b		abbreviated month name according to the current localed						"Jan" through "Dec" 	 * %B		full month name according to the current locale								"January" through "December" 	 * %c		preferred date and time representation for the current locale				"4/13/2008 12:33 PM" 	 * %C		century number (the year divided by 100 and truncated to an integer)		"00" to "99" 	 * %d		day of the month as a decimal number										"01" to "31" 	 * %D		same as %m/%d/%y															"04/13/08" 	 * %e		day of the month as a decimal number, a single digit is preceded by a space	"1" to "31" 	 * %g		like %G, but without the century											"08" 	 * %G		The 4-digit year corresponding to the ISO week number (see %V).				"2008" 	 *		This has the same format and value as %Y, except that if the ISO week number 	 *		belongs to the previous or next year, that year is used instead. 	 * %h		same as %b																	"Jan" through "Dec" 	 * %H		hour as a decimal number using a 24-hour clock.								"00" to "23" 	 * %I		hour as a decimal number using a 12-hour clock.								"01" to "12" 	 * %j		day of the year as a decimal number.										"001" to "366" 	 * %m		month as a decimal number.													"01" to "12" 	 * %M		minute as a decimal number.													"00" to "59" 	 * %n		newline character		"\n" 	 * %p		either "am" or "pm" according to the given time value, or the				"am" or "pm" 	 *		corresponding strings for the current locale. 	 * %r		time in a.m. and p.m. notation												"8:44 PM" 	 * %R		time in 24 hour notation													"20:44" 	 * %S		second as a decimal number													"00" to "59" 	 * %t		tab character																"\t" 	 * %T		current time, equal to %H:%M:%S												"12:49:11" 	 * %u		weekday as a decimal number ["1", "7"], with "1" representing Monday		"1" to "7" 	 * %U		week number of the current year as a decimal number, starting with the		"0" to ("52" or "53") 	 *		first Sunday as the first day of the first week 	 * %V		The ISO 8601:1988 week number of the current year as a decimal number,		"00" to ("52" or "53") 	 *		range 01 to 53, where week 1 is the first week that has at least 4 days 	 *		in the current year, and with Monday as the first day of the week. 	 *		(Use %G or %g for the year component that corresponds to the week number 	 *		for the specified timestamp.) 	 * %W		week number of the current year as a decimal number, starting with the		"00" to ("52" or "53") 	 *		first Monday as the first day of the first week 	 * %w		day of the week as a decimal, Sunday being "0"								"0" to "6" 	 * %x		preferred date representation for the current locale without the time		"4/13/2008" 	 * %X		preferred time representation for the current locale without the date		"12:53:05" 	 * %y		year as a decimal number without a century									"00" "99" 	 * %Y		year as a decimal number including the century								"2008" 	 * %Z		time zone or name or abbreviation											"UTC", "EST", "PST" 	 * %z		same as %Z  	 * %%		a literal "%" characters													"%" 	 * d		Day of the month, 2 digits with leading zeros								"01" to "31" 	 * D		A textual representation of a day, three letters							"Mon" through "Sun" 	 * j		Day of the month without leading zeros										"1" to "31" 	 * l		A full textual representation of the day of the week (lowercase "L")		"Sunday" through "Saturday" 	 * N		ISO-8601 numeric representation of the day of the week (added in PHP 5.1.0)	"1" (for Monday) through "7" (for Sunday) 	 * S		English ordinal suffix for the day of the month, 2 characters				"st", "nd", "rd" or "th". Works well with j 	 * w		Numeric representation of the day of the week								"0" (for Sunday) through "6" (for Saturday) 	 * z		The day of the year (starting from "0")										"0" through "365"		 	 * W		ISO-8601 week number of year, weeks starting on Monday						"00" to ("52" or "53") 	 * F		A full textual representation of a month, such as January or March			"January" through "December" 	 * m		Numeric representation of a month, with leading zeros						"01" through "12" 	 * M		A short textual representation of a month, three letters					"Jan" through "Dec" 	 * n		Numeric representation of a month, without leading zeros					"1" through "12" 	 * t		Number of days in the given month											"28" through "31" 	 * L		Whether it's a leap year													"1" if it is a leap year, "0" otherwise 	 * o		ISO-8601 year number. This has the same value as Y, except that if the		"2008" 	 *		ISO week number (W) belongs to the previous or next year, that year  	 *		is used instead. 	 * Y		A full numeric representation of a year, 4 digits							"2008" 	 * y		A two digit representation of a year										"08" 	 * a		Lowercase Ante meridiem and Post meridiem									"am" or "pm" 	 * A		Uppercase Ante meridiem and Post meridiem									"AM" or "PM" 	 * B		Swatch Internet time														"000" through "999" 	 * g		12-hour format of an hour without leading zeros								"1" through "12" 	 * G		24-hour format of an hour without leading zeros								"0" through "23" 	 * h		12-hour format of an hour with leading zeros								"01" through "12" 	 * H		24-hour format of an hour with leading zeros								"00" through "23" 	 * i		Minutes with leading zeros													"00" to "59" 	 * s		Seconds, with leading zeros													"00" through "59" 	 * u		Milliseconds																"54321" 	 * e		Timezone identifier															"UTC", "EST", "PST" 	 * I		Whether or not the date is in daylight saving time (uppercase i)			"1" if Daylight Saving Time, "0" otherwise 	 * O		Difference to Greenwich time (GMT) in hours									"+0200", "-0600" 	 * P		Difference to Greenwich time (GMT) with colon between hours and minutes		"+02:00", "-06:00" 	 * T		Timezone abbreviation														"UTC", "EST", "PST" 	 * Z		Timezone offset in seconds. The offset for timezones west of UTC is			"-43200" through "50400" 	 *			always negative, and for those east of UTC is always positive. 	 * c		ISO 8601 date																"2004-02-12T15:19:21+00:00" 	 * r		RFC 2822 formatted date														"Thu, 21 Dec 2000 16:01:07 +0200" 	 * U		Seconds since the Unix Epoch (January 1 1970 00:00:00 GMT)					"0" 	 * @param {String}   A format string consisting of one or more format spcifiers [Optional]. 	 * @return {String}  A string representation of the current Date object. 	 */ 	var formatReplace = function (context) { 		return function (m) { 			var formatString, override = false; 			Iif (m.charAt(0) === "\\" \|\| m.substring(0, 2) === "%%") { 				return m.replace("\\", "").replace("%%", "%"); 			}   			override = normalizer.shouldOverrideDefaults(m); 			formatString = $D.normalizeFormat(m, context); 			Eif (formatString) { 				return context.toString(formatString, override); 			} 		}; 	}; 	$P._format = function (format) { 		var formatter = formatReplace(this); 		if (!format) { 			return this._toString(); 		} else { 			return format.replace(/(%\|\\)?.\|%%/g, formatter); 		} 	};   	Eif (!$P.format) { 		$P.format = $P._format; 	} }()); ``` |
```

Generated by istanbul at Sun Nov 16 2014 12:08:33 GMT-0800 (PST)
